# Risk Factors Associated with Poor Computer-based Testing (CBT) Scores - Comparing Students' Performance “Without/With COVID-19” and Backgrounds –

**DOI:** 10.14789/jmj.JMJ21-0037-OA

**Published:** 2022-02-16

**Authors:** YUICHI TOMIKI, MOTOMI NASU, AMANE ENDO, MIWA SEKINE, MAKINO WATANABE, HIROO WADA, YUJI NISHIZAKI, TSUTOMU SUZUKI, TAKAO OKADA

**Affiliations:** 1 Division of Medical Education, Juntendo University Faculty of Medicine, Tokyo, Japan; 1 Division of Medical Education, Juntendo University Faculty of Medicine, Tokyo, Japan; 2 Department of Coloproctological Surgery, Juntendo University Faculty of Medicine, Tokyo, Japan; 2 Department of Coloproctological Surgery, Juntendo University Faculty of Medicine, Tokyo, Japan; 3 Department of Esophageal and Gastroenterological Surgery, Juntendo University Faculty of Medicine, Tokyo, Japan; 3 Department of Esophageal and Gastroenterological Surgery, Juntendo University Faculty of Medicine, Tokyo, Japan; 4 Department of Pediatrics and Adolescent Medicine, Juntendo University Faculty of Medicine, Tokyo, Japan; 4 Department of Pediatrics and Adolescent Medicine, Juntendo University Faculty of Medicine, Tokyo, Japan; 5 Department of Microbiology, Juntendo University Faculty of Medicine, Tokyo, Japan; 5 Department of Microbiology, Juntendo University Faculty of Medicine, Tokyo, Japan; 6 Department of Organ and Cell Physiology, Juntendo University Faculty of Medicine, Tokyo, Japan; 6 Department of Organ and Cell Physiology, Juntendo University Faculty of Medicine, Tokyo, Japan; 7 Department of Public Health, Juntendo University Faculty of Medicine, Tokyo, Japan; 7 Department of Public Health, Juntendo University Faculty of Medicine, Tokyo, Japan; 8 Department of General Medicine, Juntendo University Faculty of Medicine, Tokyo, Japan; 8 Department of General Medicine, Juntendo University Faculty of Medicine, Tokyo, Japan; 9 Department of Cardiovascular Biology and Medicine, Juntendo University Faculty of Medicine, Tokyo, Japan; 9 Department of Cardiovascular Biology and Medicine, Juntendo University Faculty of Medicine, Tokyo, Japan; 10 Department of Respiratory Medicine, Juntendo University Faculty of Medicine, Tokyo, Japan; 10 Department of Respiratory Medicine, Juntendo University Faculty of Medicine, Tokyo, Japan

**Keywords:** CBT, COVID-19, online lecture, self-learning, extracurricular activity

## Abstract

**Objective:**

The present study compared students' CBT scores during the 2-year period before (“without COVID-19”) and 2-year period during (“with COVID-19”) the COVID-19 pandemic, and analyzed factors associated with poor results.

**Materials:**

A total of 530 students (368 males and 162 females), who had taken CBT within the period between 2018 and 2021.

**Methods:**

Analysis was performed based on the questionnaire results, and the students' performance was compared between “without/with COVID-19” to identify the causes of poor CBT scores.

**Results:**

The overall mean IRT score was 515.5±85.4. The without and with COVID-19 groups' scores were 495.7±85.9 and 534.4±80.8, respectively (p<0.01). Among all students, 43 (8.1%) had IRT scores lower than 400 as poor CBT scores; 27 (10.4%) without and 16 (5.9%) with COVID-19, revealing a decrease in the latter. The multivariate analysis of the risk of students having poor CBT scores showed that students with poor performance during the third year (odds ratio:7.02), starting preparation for CBT late (2.19), and not taking any practice examination (4.58) are more likely to have poor CBT results.

**Conclusions:**

Due to the COVID-19 pandemic, students spent more time on online home study, and this may have consequently improved their CBT scores. Such learning performance is desirable for medical students, but they have lost the opportunity to gain valuable experiences that they could have acquired through extracurricular activities, such as club activities. In this respect, we cannot simply be pleased by the improvement in students' CBT scores.

## Introduction

The rule that requires students to pass 2 common achievement tests: computer-based testing (CBT) and Objective Structured Clinical Examination (OSCE), in order to participate in clinical exercises is widely recognized among medical schools throughout Japan. Questions presented in CBT vary among examinees, but the level of difficulty is adjusted based on the item response theory (IRT). As the passing IRT score is fixed at 359 or higher, students with IRT scores lower than this in the main test and re-test are bound to repeat a year at this point. Therefore, students study hard to pass these tests^1)^.

Based on the model core curriculum, CBT examines basic knowledge of basic medicine, clinical medicine, and social medicine, and, serving as a mid-term examination during the 6-year medical school period, it is called “the Pre-national Medical Practitioners Qualifying Examination”. As the Medical Practitioners' Law was revised at last, it has been decided to define common achievement tests (CBT and OSCE) before clinical exercise as public examinations from 2023.

The coronavirus disease (COVID-19), which began to globally spread from 2019, has negatively affected not only people's health, but also many fields, including economic activities. Among medical institutions, there have been fears of a collapse of healthcare services due the chaos in hospitals caused by this unknown infectious disease, while concerns over a decline in academic performance have been raised at educational institutions due to the prolonged closure of schools. The suspension of educational activities also has had a significant impact on medical schools, which were forced to adopt measures for students to attend lectures and exercises at home, mainly using online systems^2, 3)^. It is necessary to clarify how medical students in such a special situation have learned and taken CBT.

In our previous studies, we reported that “students who have taken a practice examination achieve better CBT scores than those who have not”, and “students who selected biology for the university entrance examination achieved better CBT scores than those who selected physics^4-6)^. Preparation for CBT requires regular effort, but, needless to say, it is better for students to start such preparation earlier. Furthermore, as CBT is conducted immediately after the summer vacation for the fourth year in our university, the way to spend this period is the important point.

The present study compared students' CBT scores during the 2-year period before (“without COVID-19”) and 2-year period during (“with COVID-19”) the COVID-19 pandemic, and analyzed factors associated with poor results. As students with poor CBT scores are likely to also face difficulties in the subsequent graduation examination and national qualification examination, it aims to provide a useful insight for guiding students toward the establishment of suitable study methods through preparation for CBT during the fourth year^6)^.

## Materials and Methods

Subjects: A total of 541 students (377 males and 164 females), who had taken CBT within the period between 2018 and 2021.

Methods: A questionnaire survey on preparation for CBT was conducted after it.

The questionnaire consisted of the following questions:

1) What is your science elective for your university entrance examination?

2) What were your grades in the third year? (self- report)

3) When did you start preparing for CBT?

4) Did you participate in the summer camp as a club activity?

5) Did you have a part-time job during the summer vacation?

6) Did you travel during the summer vacation?

7) Have you taken any practice examination for CBT?

Excluding 5 with no answers to any question and 6 who had taken the examination, selecting “physics-biology”, 530 students (368 males and 162 females) were examined.

Their IRT scores for CBT were classified into 6 grades: 1: lower than 300, 2: 300-399, 3: 400-499, 4: 500-599, 5: 600-699, and 6: 700 or higher. In the present study, IRT scores lower than 400 (Grades 1 and 2) were defined as poor CBT scores.

Analysis was performed based on the questionnaire results, and the students' performance was compared between “without/with COVID-19” to identify the causes of poor CBT scores.

All values are shown as the mean ± SD (range). Statistical analysis was performed using t-test and chi-square test, in addition to JMP 9.0 (SAS Institute Inc., Cary, NC, USA). The significance level was set at p<0.05 in all settings.

Data regarding the students' results were processed, adopting measures to prevent the identification of individuals, and their written consent to the use of these data for research purposes was previously obtained. The present study was conducted with the approval of the ethics committee of Juntendo University (Approval number: 2018129).

## Results

## 1. Comparison of performance between the without/with COVID-19 groups

**Figure 1:**
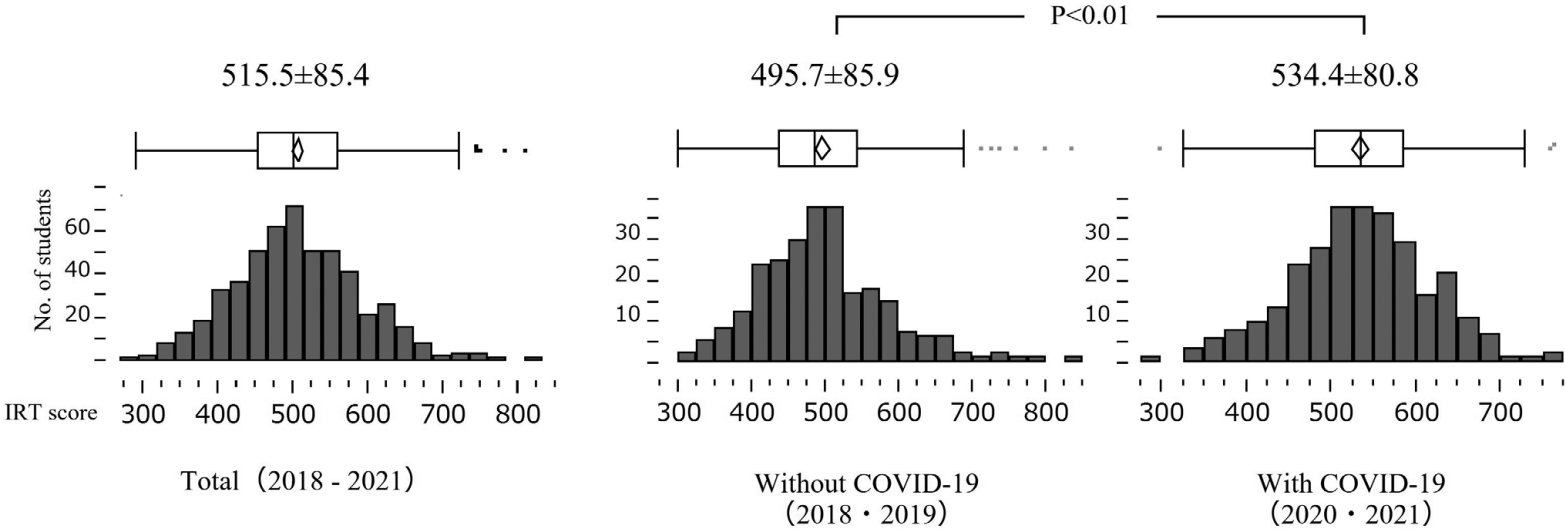
Overall mean IRT score and the Without/With COVID-19 groups' IRT scores

The overall mean IRT score was 515.5±85.4. The without and with COVID-19 groups' scores were 495.7±85.9 and 534.4±80.8, respectively. Thus, the latter's score was significantly higher (p<0.01) (Figure 1).

On comparing the number of students at each grade based on their IRT scores, there were more students at Grades 4 and 5 and fewer students at Grade 3 in the with than without COVID-19 group (p<0.01 in both cases). Furthermore, among all students, 43 (8.1%) had IRT scores lower than 400 as poor CBT results; 27 (10.4 %) without and 16 (5.9 %) with COVID-19, revealing a decrease in the latter (Table 1).

**Table 1:** Number of students at each grade

IRT score	Grade	Total (n=530)	Without COVID-19 (n=259)	With COVID-19 (n=271)	P-value
≧ 700	6	10 ( 1.9%)	6 ( 2.3%)	4 ( 1.5%)	P＝0.48
600 - 699	5	72 (13.6%)	21 ( 8.1%)	51 (18.8%)	P＜0.01
500 - 599	4	219 (41.3%)	88 (34.0%)	131 (48.3%)	P＜0.01
400 - 499	3	186 (35.1%)	117 (45.2%)	69 (25.5%)	P＜0.01
＜400	2, 1	43 ( 8.1%)	27 (10.4 %)	16 ( 5.9 %)	P＝0.06

## 2. Science electives and performance during the third year

On comparing performance between the sexes, females' scores were higher. The rate of students with poor CBT scores was higher among males of the without COVID-19 group and among females of the with COVID-19 group.

The students were further divided into physics and biology groups, irrespective of their science electives for university entrance examinations. The without COVID-19/physics group's IRT score was 484.7±85.0, which was significantly lower than the 515.1±84.3 of the without COVID-19/biology group (p<0.01), and the number of members with poor CBT scores was as large as 21 (12.3%). In the with COVID-19 group, there were no differences related to science electives (Table 2).

**Table 2:** Differences related to sex and science electives

		Without COVID-19				With COVID-19		
		No. of students	IRT score	No. of Poor CBT scores		No. of students	IRT score	No. of Poor CBT scores
Sex								
	Male	181	495.2±91.4	22 (12.2%)		187	529.9±79.8	9 (4.8%)
	Female	78	497.0±72.0	5 (6.4%)		84	544.5±82.5	7 (8.3%)

Science electives for university entrance examinations								
	Physics Group	165	484.7±85.0^‡^	21 (12.3%)		171	532.9±92.1	8 (4.7%)
	Biology Group	94	515.1±84.3	6 (6.4%)		100	536.9±73.7	8 (8.0%)

‡P<0.01

As for the students' self-reported performance in their third year, those with poorer performance during this period had lower IRT scores. It was particularly noted that the IRT scores of many students realizing that they were among the bottom quartile were poor. Specifically, 22 (25.3%) of the without and 13 (15.9%) of the with COVID-19 group had poor CBT scores (Table 3).

**Table 3:** Performance in the third year (Self-reported)

	Without COVID-19				With COVID-19		
Grades in the third year (Self-reported)	No. of students	IRT score	No. of Poor CBT scores		No. of students	IRT score	No. of Poor CBT scores
1~25％	26	629.7±85.9^‡^	0		40	616.5±56.0^‡^	0
25～50％	65	527.2±62.2^‡^	2(3.1%)		71	572.4±62.3^‡^	1(1.4%)
50～75％	81	493.2±60.6^‡^	3(3.7%)		78	530.7±58.6^‡^	2(2.6%)
75～100％	87	434.5±60.9	22(25.3%)^‡^		82	464.9±64.3	13(15.9%)^‡^

‡P<0.01

## 3. The time of starting preparation for CBT

On comparing the time of starting preparation for CBT, the IRT scores of students who had started their preparation from 6 months in advance were higher, and the score decreased with a 1-month delay in starting. Among students who had started it 3 months in advance, the rate of having a poor CBT score was higher in both the without and with COVID-19 groups, 15.5 and 15.9%, respectively, and the difference was significant in the latter (p<0.01).

In the without COVID-19 group, 25% of all students who had started preparing for CBT 1 month in advance had poor CBT scores (p=0.0142) (Table 4).

**Table 4:** Time of starting preparation for CBT

	Without COVID-19				With COVID-19		
Time of starting preparation	No. of students	IRT score	No. of Poor CBT scores		No. of students	IRT score	No. of Poor CBT scores
More than 6 months in advance	25	534.2±94.2	1(4.0%)		35	575.9±80.6	1(2.9%)
5 months in advance	58	512.5±77.2	3(5.2%)		70	559.1±67.3	1(1.4%)
4 months in advance	45	489.4±74.4	4(8.9%)		42	548.7±84.2	2(4.8%)
3 months in advance	58	488.1±98.5	9(15.5%)		55	501.9±85.9	8(15.9%)^‡^
2 months in advance	49	494.4±82.8	4(8.2%)		43	504.7±68.2	2(4.7%)
1 month in advance	24	448.1±69.3	6(25.0%)^†^		26	506.5±68.4	2(7.7%)

†P<0.05, ‡P<0.01

Combining the students' performance in their third year and the time they had started preparing for CBT, it was found that the time was earlier among students with favorable results and later among those with poor results. Among 196 students realizing that they were upper lanked, 135 (68.9%) had started their preparation for CBT 4 months in advance (Table 5: light gray area). In contrast, among 328 realizing that they had a low rank, 188 (57.3%) had started it 3 months in advance (Table 5: dark gray area). Especially, 14.8% of all students realizing that they were among the bottom quartile had started it 1 month in advance, which was after the beginning of the summer vacation (Table 5).

**Table 5:**
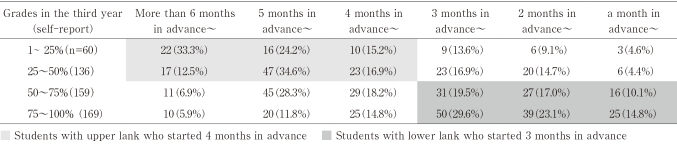
Relationship between performance in the third year and the time of starting preparation for CBT

On comparing the time of starting preparation in each science-elective-based group, the rate of students starting it more than 4 months in advance did not vary between without and with COVID-19 in the biology group, whereas there was an increase of 8.3% with COVID-19 in the physics group (Table 6).

**Table 6:** Time of starting preparation for CBT in each science-elective-based group

	Without COVID-19				With COVID-19		
		Time of starting preparation				Time of starting preparation	
	No. of students	more than 4 months in advances	3 months in advances		No. of students	more than 4 months in advances	3 months in advances
Physics Group	165	75 (45.5%)	90 (54.5%)		171	92 (53.8%)	79 (46.2%)
Biology Group	94	53 (56.4%)	41 (43.6%)		100	55 (55.0%)	45 (45.0%)

## 4. The ways to spend the summer vacation

In the with COVID-19 group, there were no students who had participated in the summer camp, whereas 170 of the without COVID-19 group had participated in it, and their IRT scores were significantly lower than those of the former (p=0.03).

Furthermore, in the without COVID-19 group, the IRT score tended to be low when the sum of the days spent participating in the summer camp, working part time, and travelling was 10 or more (p=0.04).

The IRT scores of students who had not taken any practice examination were significantly lower in both the without and COVID-19 groups (p<0.01, p=0.01), and the rate of having a poor CBT score was significantly higher among these students (p<0.01) (Table 7).

**Table 7:** Ways to spend the summer vacation

		Without COVID-19				With COVID-19		
		No. of students	IRT score	No. of Poor CBT scores		No. of students	IRT score	No. of Poor CBT scores
a) Summer camp	Yes	170	486.5±73.3^†^	17(10.0%)		0	－	
	No	89	513.3±104.1	10(11.2%)		271	534.4±80.8	16(5.9%)
b) Working part time	Yes	110	489.9±76.7	11(10.0%)		118	534.2±78.8	7(5.9%)
	No	149	500.0±88.7	16(10.7%)		153	534.5±82.6	9(5.9%)
c) Travelling	Yes	78	507.5±78.2	4(5.1%)		38	527.9±71.7	1(2.6%)
	No	181	490.6±87.3	23(12.7%)		233	535.4±82.3	15(6.4%)
Summer vacation events (a + b + c)	≥10 days	135	485.3±78.3^†^	15(11.1%)		30	522.3±89.5	4(13.3%)
	< 10 days	124	507.1±92.4	12(9.7%)		241	535.9±79.7	12(5.0%)
Practice examination	Yes	132	519.3±90.2	7(5.3%)		258	538.3±77.7^†^	11(4.3%)
	No	127	471.3±73.9^‡^	20(15.8%)^‡^		13	456.3±102.3^†^	5(38.5%)^‡^

†P<0.05, ‡P<0.01

## 5. Multivariate analysis to identify risk factors associated with poor CBT scores

With <science elective (biology vs. physics)>, <performance in the third year (upper half group vs. lower half group)>, <the time of starting preparation (4 months in advance vs. 3 months in advance or later)>, <participation in the summer camp (not participating vs. participating)>, <the sum of the days spent having special summer events (less than 10 vs. 10 or more)>, and < practice examination (taking vs. not taking)> that revealed significant differences in univariate analysis as covariances, multivariate analysis was performed to identify risk factors associated with poor CBT scores.

Then, <performance in the third year (odds ratio: 7.02)>, <the time of starting preparation (2.19)>, and < practice examination (4.58)> were identified as such factors (Table 8). Thus, it was shown that students with poor performance in the third year, starting preparing for CBT late, and not taking any practice examination are more likely to have poor CBT scores.

**Table 8:** Multivariate analysis to identify risk factors associated with poor CBT scores

	regression coefficient	odds ratio	95% confidence interval	P-value
Science electives for university entrance examinations (Biology Group vs Physics Group)	0.01	1.02	0.49 - 2.05	0.95
Grades in the third year (Upper half Group vs Lower half Group)	0.96	7.02	2.43 - 29.7	＜0.01^‡^
Time of starting preparation (More than 4 months vs 3 months in advances)	0.39	2.19	1.09 - 4.68	0.03^†^
Summer camp (Participating Group vs Not participating Group)	0.28	1.75	0.76 - 4.14	0.19
Summer vacation events (<10 days vs ≥10 days)	0.26	1.67	0.71 - 3.17	0.20
Practice examination (Taken vs Not taken)	0.76	4.58	2.24 - 9.52	＜0.01^‡^

†P<0.05, ‡ P<0.01

## Discussion

Clearly insufficient study and a superficial understanding lead to failures in CBT, and clearly insufficient study results from a late start of preparation. When running out of time for preparation, students begin to simply resolve practice questions, rather than studying, which results in superficial knowledge without a deep understanding as a vicious circle. Students with poorer performance need to start their preparation earlier^4, 5)^.

The clear difference between the without and with COVID-19 groups in this study comes from the difference between online and home learning. The without COVID-19 group started their preparations for regular tests and CBT, while attending face-to-face lectures and exercises and engaging in extracurricular activities, such as club activities and part-time jobs. During the summer vacation, they participated in the summer camp for the East Japan Sports Meeting of Medical Students (“Toitai”), and then needed to concentrate on their preparation for CBT. Thus, they spent a productive summer, but with no margin of time. In contrast, the with COVID-19 group experienced a switch of face-to face lectures and exercises to home learning, mainly online, and their extracurricular activities, such as club activities and part-time jobs, were restricted. Accordingly, the time they spent at home overwhelmingly increased. Not being allowed to go out, they had no choice but to devote this time to study. With the Toitai also cancelled and no summer camp, they were able to use most of the summer vacation to prepare for CBT. Allocating much time to study at home, they may have achieved improved CBT scores^7)^. When the education curriculum and extracurricular activities are gradually resumed in the future, students will not have as much time to prepare for CBT as they did this time, but the important thing is to make a study schedule, and stick to it with a sense of urgency.

Moreover, medical students nowadays not only have more time to study, but they are also likely to be more proficient in study methods using online systems. With regard to the acquisition of knowledge, face-to-face lectures in classrooms are not necessarily required, and the results of the present study may have demonstrated that online lectures and self-learning using online systems suffice for students to acquire knowledge to the level they can pass CBT^8)^. There is no doubt that this pandemic has been a turning point to reform medical education. Specifically, online education, which had not been widely adopted up until it, has been rapidly disseminated throughout Japan. In the early stages of the COVID-19 pandemic, online lectures were an alternative to face-to-face lectures, but now they are recognized as one of the most important educational tools. The introduction of online lectures, especially on-demand lectures, has greatly changed medical students' learning styles, and such lectures are likely to continue to be favored by students. In the future, it is necessary to review face-to-face lectures in classrooms, while expanding educational approaches using online systems^9)^. In such an unprecedented and uncertain situation, medical students' efforts to pass CBT and achieve better scores than before the pandemic are commendable.

In the without COVID-19 group, the rate of having a poor CBT score was higher among males, indicating that some male students prioritize their extracurricular activities such as club activities, rather than study, and do not sufficiently study^10)^. In addition, many questions in CBT are based on biology, and students who choose physics are disadvantaged to some extent compared to those who choose biology in their entrance examinations. We previously reported a higher rate of choosing physics among male students, which is one of the reasons^5)^. However, during the COVID-19 pandemic, even male students who had been prioritizing their extracurricular activities had enough time to study, and in the physics groups, an increased number of students started to prepare for CBT early. Therefore, neither the sex nor science elective caused differences.

CBT examines basic knowledge of basic medicine, clinical medicine, and social medicine. As these areas are extensively learned, it is natural that students' performance up to the third year affects their CBT scores, and it is obvious that regular effort is more important than last-minute study. Although it is difficult to prepare for CBT in parallel with study for the regular curriculum and regular tests, it is important for students to plan their study, focusing on CBT, from the beginning of the fourth year, and start preparing for it at least 4 months in advance. Realizing one's poor performance, but preparing late for an examination that your promotion depends on indicates a lack of urgency and the necessity of reviewing your attitude toward the examination in the future.

As the CBT is conducted immediately after the summer vacation in our university, how to spend this period is the important point. In the without COVID-19 group, the IRT scores of students who participated in the summer camp were significantly lower than the scores of those who did not, suggesting that students do not study during the camp even if they bring their own study tools. The IRT scores of students who spent more than 10 days for events other than study, such as the summer camp, were also lower, but this may be attributed in large part to the performance of those who had participated in the summer camp. In any case, students had better give priority to preparation for CBT during the summer vacation in their fourth year.

Most students prepare for CBT using the workbooks published by a publisher specializing in medicine, which have recently been shifted from paper-based to online. Additionally, purchasers of the workbook, who have registered with an online membership system, are allowed to take a practice examination for free as a special benefit. Our university does not force students to take a practice examination, but based on data showing that the performance of students taking practice examinations is better than that of students not taking them, it has recently begun to recommend that students take them^4)^. Accordingly, most of the with COVID-19 group had taken practice examinations. The better performance of these students may have resulted from leeway for preparation and the ability to objectively assess their own achievement level and overcome their weak points, acquired by taking practice examinations^4)^. The without COVID- 19 group's reason for not taking any practice examination may have been because they did not have enough time to take it, or they studied using paper-based questions collections handed down from senior students. The with COVID-19 group's reason for not taking it is unclear, but it is possible that mental instability made it difficult for them to concentrate on study^11)^.

The multivariate analysis of the risk of students having poor CBT scores showed that students with poor performance during the third year, starting preparation for CBT late, and not taking any practice examination are more likely to have poor CBT results. Based on this, students with poorer performance need to start preparing for CBT earlier. As previously mentioned, the with COVID-19 group's reason for not taking any practice examination is unclear, but this will not be the cause of poor CBT results anymore in the future, as taking such practice examinations will be standard.

As a limitation of this study, the results were obtained only by analyzing a single facility. Additionally, students' performance was compared between without and with the COVID-19 pandemic, the latter of which is an unprecedented and special situation. Some students had their days and nights reversed due to not commuting to school, which resulted in an unhealthy circadian rhythm, while others' mental health became unstable due to living alone and not being able to return home or go back to their countries. Therefore, the possibility of these students having been unable to show their true potential should be taken into account^11)^. It is interesting to know whether students' CBT performance will return to the previous level “after COVID-19”, when the situation is settled, and extracurricular activities are resumed. We will continue to follow up on this. We should also examine some problems caused by the suspension of extracurricular activities due to the COVID-19 pandemic, as such activities, including club activities, are important in student life. Helping students restore and maintain their motivation that has decreased due to the loss of goals is a top priority. Supplementary approaches for the development of communication skills in interpersonal relationships and the acquisition of a regular life and common sense behaviors during their remaining days at university are also required. We will continue to monitor this generation, whose student lives were affected by the COVID-19 pandemic, to clarify its influences on their motivation and personality development in the future.

Due to the COVID-19 pandemic, students spent more time on online home study, and this may have consequently improved their CBT scores. Such learning performance is desirable for medical students, but they have lost the opportunity to gain valuable experiences that they could have acquired through extracurricular activities, such as club activities. In this respect, we cannot simply be pleased by the improvement in students' CBT scores.

## Funding

The authors received no financial support for the research.

## Author contributions

This manuscript was drafted by YT, TS, and TO. The questionnaire was compiled by MW and MS. Data entry was done by MN and AE, and data interpretation and statistical analysis were done by HW. YT and YN contributed significantly to the writing of this manuscript. All authors read and approved the final manuscript.

## Conflicts of interest statement

The Authors declares that there are no conflicts of interest.
